# Unilateral Biportal Endoscopic Spine Surgery: Managing Complex and Revision Cases With a Minimally Invasive Approach

**DOI:** 10.7759/cureus.99815

**Published:** 2025-12-22

**Authors:** Alhareth Maaya, Jin Hwa Eum

**Affiliations:** 1 Surgery, Ain Al Khaleej Hospital, Abu Dhabi, ARE

**Keywords:** cervical spine revision, lumbar spine revision, spine, thoracolumbar pathology, ube spine surgery, unilateral biportal endoscopic, vas score, visual analog scale

## Abstract

Revision spinal surgeries present challenges due to complications from previous surgeries, such as scar tissue and altered anatomy. Unilateral biportal endoscopic (UBE) spine surgery is a versatile, minimally invasive technique that can effectively address a wide range of spinal pathologies, including complex and revision cases. This case series aimed to evaluate the safety and efficacy of UBE spine surgery in managing complex and revision spinal cases by retrospectively reviewing three challenging cases managed with UBE surgery. These included a cervical spine revision, a lumbar spine revision, and a complex thoracolumbar pathology in an elderly patient. Each case was evaluated for clinical outcomes, including visual analog scale (VAS) scores, neurological function, and overall patient satisfaction. Case 1 involved a 45-year-old female patient with persistent radicular pain after anterior cervical discectomy and arthroplasty. UBE decompression resulted in complete pain relief (VAS 8/10 to 0/10) at the two-year follow-up. Case 2 was of a 49-year-old male patient with cauda equina syndrome post-L5/S1 posterior lumbar interbody fusion (PLIF), who showed marked improvement in left foot power (0/5 to 4/5) and reduced VAS scores (9/10 to 1/10) at the six-month follow-up. Case 3 involved a 71-year-old female patient with pathological fractures and multiple comorbidities, where UBE cord decompression, kyphoplasty, and screw fixation performed under local anesthesia resulted in significant pain relief (VAS 0-1/10) and improved mobility at the two-year follow-up. This case series showcases the versatility of UBE surgery in managing complex and revision spinal cases, with excellent clinical outcomes, especially in patients with significant comorbidities.

## Introduction

The growing global population with increased life expectancy is driving a surge in cases of degenerative spinal diseases [1]. An increasing list of medical comorbidities, such as liver, lung, heart, and kidney disease, as well as the increased hazards of general anesthesia, needs to be addressed by surgeons as patients get older. The usage of endoscopy-based spinal surgeries is rising as a result of a growing percentage of elderly patients preferring minimally invasive surgical procedures compared to traditional surgical options. Spinal surgeries, particularly revision procedures, are often complex due to the presence of scar tissue, altered anatomy, and potential complications from previous interventions. Revisions are more difficult than main procedures because of changed anatomical landmarks, avascular scars from prior surgery, and epidural sclerosis [2]. Most available studies on primary versus revision spine surgery outcomes are limited by small sample sizes, which may affect the generalizability of their findings. [3-5]. Another study revealed that Medicare recipients with multiple health conditions faced elevated risks when undergoing revision instrumented fusion procedures [6].

Traditional open spine surgeries, while effective, carry significant risks and longer recovery times. These procedures often require extensive approaches and may be performed in multiple stages to manage the intricate anatomical challenges and ensure a comprehensive treatment of the pathology. However, such extensive surgeries are associated with a high incidence of complications. Endoscopic surgery, a sub-branch of minimally invasive spinal surgery, is undergoing rapid development to provide safer and more effective treatment options for older patients susceptible to the risks of general anesthesia [7,8].

Unilateral biportal endoscopic (UBE) spine surgery has gained increasing attention as a minimally invasive technique, offering reduced tissue disruption, less postoperative pain, and faster recovery, though some aspects of its use in complex cases remain under investigation. [9]. This minimally invasive technique involves the use of two portals, allowing for a broader working area and better visualization compared to traditional endoscopic methods. The versatility of UBE makes it suitable for a wide range of spinal pathologies, including complex and revision cases, while minimizing tissue disruption and preserving anatomical structures. In a study on biportal endoscopic revision surgery for adjacent segmental disease (ASD) after lumbar arthrodesis, Zhu et al. presented three cases of ASD with radiculopathy in patients aged 56, 67, and 78, all with a history of L4 to L5 fusion [10]. Using UBE decompression, they successfully treated conditions like spinal epidural lipomatosis, up-migrated disc herniation, and foraminal stenosis, and postoperative outcomes showed significant clinical improvement and nerve root decompression with no complications. In another study, Gao et al. evaluated UBE-assisted transforaminal lumbar interbody fusion (TLIF) for recurrent lumbar disc herniation (RLDH) in 44 patients [11]. They found UBE-TLIF effective, with minimal blood loss, shorter recovery times, and improved pain and functional scores. The technique showed high patient satisfaction and is considered a safe, minimally invasive treatment for RLDH.

The literature indicates that the complication rate for revision spine surgeries can be as high as 25-30%, with increased risks of dural tears, infections, neurovascular injuries, and prolonged hospital stays [9,11-13]. Furthermore, complex primary surgeries, such as those addressing deformities or significant degenerative changes, also present a high complication rate, often ranging 20-25%. These complications contribute to extended recovery times, increased healthcare costs, and a substantial impact on patients' quality of life [12,14]. The advantages of UBE spine surgery include reduced surgical morbidity, minimized blood loss, and shorter recovery times compared to traditional open surgery. The technique allows for precise decompression, discectomy, and fusion procedures with less trauma to the surrounding tissues. Additionally, UBE can be performed under local anesthesia and sedation, which is particularly beneficial for patients with significant comorbidities who are at high risk for complications associated with general anesthesia.

This case series aims to present our experience of the safety, efficacy, and patient outcomes of UBE spine surgery in managing complex and revision spinal cases. It highlights the benefits of UBE, including its effectiveness in treating complex spinal diseases, reducing surgical trauma, and improving recovery times. This series as it addresses the growing need for minimally invasive spinal surgery options, mainly for elderly patients with multiple comorbidities. It underscores the potential of UBE spine surgery in reducing surgical morbidity and blood loss, and shortening recovery times compared to traditional techniques. By presenting successful outcomes in complex and revision cases, this report aims to advocate for UBE as a viable alternative to conventional spine surgeries, emphasizing its ability to offer high-quality, patient-centered care with fewer complications and positive clinical results.

## Case presentation

This was a retrospective review of three challenging cases managed using UBE spine surgery. The cases were selected based on the complexity of the pathology, the presence of significant comorbidities, and the previous surgical interventions [9,15]. The patients included in this series presented with complex spinal diseases or required revision surgery due to continuous or repetitive symptoms after prior spinal surgeries. The inclusion criteria were the presence of persistent or recurrent symptoms despite prior spinal surgery, X-ray, CT, and MRI examinations of disease demanding surgery, and a preference for a minimally invasive approach due to comorbidities or previous complications from open surgeries.

Preoperative assessment

Each patient underwent a thorough preoperative evaluation, which included a detailed clinical examination, pain assessment using the Visual Analog Scale (VAS) [16], and a thorough neurological examination. Imaging studies, such as X-ray, MRI, and CT scans, were conducted to assess the extent of spinal pathology and guide surgical planning. The patients' medical histories and previous surgical records were reviewed to tailor the surgical approach and anticipate potential challenges accordingly.

Surgical method

The UBE technique was used to manage complex spinal diseases. The procedure began with the patient being placed in the prone position on a radiolucent operating table under general anesthesia or local anesthesia with sedation, depending on the patient's comorbidities and preferences. An X-ray fluoroscopy machine was used to determine the precise location of the surgical site, followed by routine disinfection and draping. Two small skin incisions, strategically placed to optimize access to the disease while minimizing tissue disruption, served as the portals for endoscopic surgery. The first portal was designed for the endoscope, providing continuous visualization of the surgical field, while the second portal was used to insert surgical instruments [15,17]. The positions of the portals were confirmed using fluoroscopy. Once the portals were in place, the endoscopic procedure commenced. The target area was identified and exposed using plasma electronic burning. The surgical steps involved the removal of the hypertrophic ligamentum flavum and any herniated disc material to decompress the affected spinal segments. In cases involving revision surgeries, displaced hardware or interbody cages were carefully removed under endoscopic guidance [18].

In cases requiring stabilization, we performed endoscopic TLIF or kyphoplasty, with fluoroscopic guidance ensuring proper implant placement [19,20]. Additionally, percutaneous transpedicular screw fixation was performed as needed to provide further stabilization [21]. The surgery was completed by ensuring proper positioning of all instrumentation through intraoperative fluoroscopic guidance. The incisions were closed with subcutaneous sutures and covered with a sterile dressing. Postoperative care included standard protocols to ensure optimal recovery and follow-up assessments to monitor the patients' progress.

Postoperative care

After the UBE spine surgery, all suspected bleeding points were carefully monitored, and a drain was placed to manage any postoperative fluid accumulation. Pain management was addressed with a combination of analgesics tailored to individual needs, and a structured early mobilization protocol was initiated. On the second postoperative day, patients were advised to use a support belt and to begin mobilization as soon as possible to enhance functional recovery. Physiotherapy sessions were scheduled to aid in rehabilitation and improve overall mobility. We aimed to arrange regular follow-up visits at one month, six months, and two years postoperatively, with variations depending on patient condition and compliance [16]. The assessment included the evaluation of pain relief using the VAS, neurological function through motor and sensory examination, and radiographic imaging to check the positioning of hardware and fusion status. Patient satisfaction and overall quality of life (QOL) were also assessed to gauge the long-term effectiveness of the UBE approach. Postoperative care included standard pain management, early mobilization, and physiotherapy. If possible, we tried to address complications and arrange regular follow-up visits to assess clinical outcomes.

Cases

Case 1: Cervical Spine Revision Surgery

A 45-year-old female patient presented with persistent right-sided radicular pain (VAS 8/10) and axial neck pain. She had a history of anterior cervical discectomy and total disc replacement (TDR) at C5/C6 performed at another institution two years prior to presentation. Despite initial surgical intervention, her symptoms remained refractory. Preoperative MRI, although limited by significant metal artifact from the implant, demonstrated compression at the C5/C6 level. Preoperative CT imaging was not available as the patient underwent her initial surgery abroad and currently resides outside our region, limiting access to additional imaging.

Given the imaging limitations and the clinical picture, posterior or foraminal pathology was considered the most likely cause of her ongoing symptoms. A definitive anterior revision procedure involving TDR removal and anterior cervical discectomy and fusion (ACDF) was discussed with the patient. However, she declined further anterior surgery, expressing a preference for a minimally invasive approach. With documented informed consent, a decision was made to proceed with posterior cervical decompression and discectomy using the UBE technique as a bridging, symptom-relieving intervention. 

**Figure 1:**
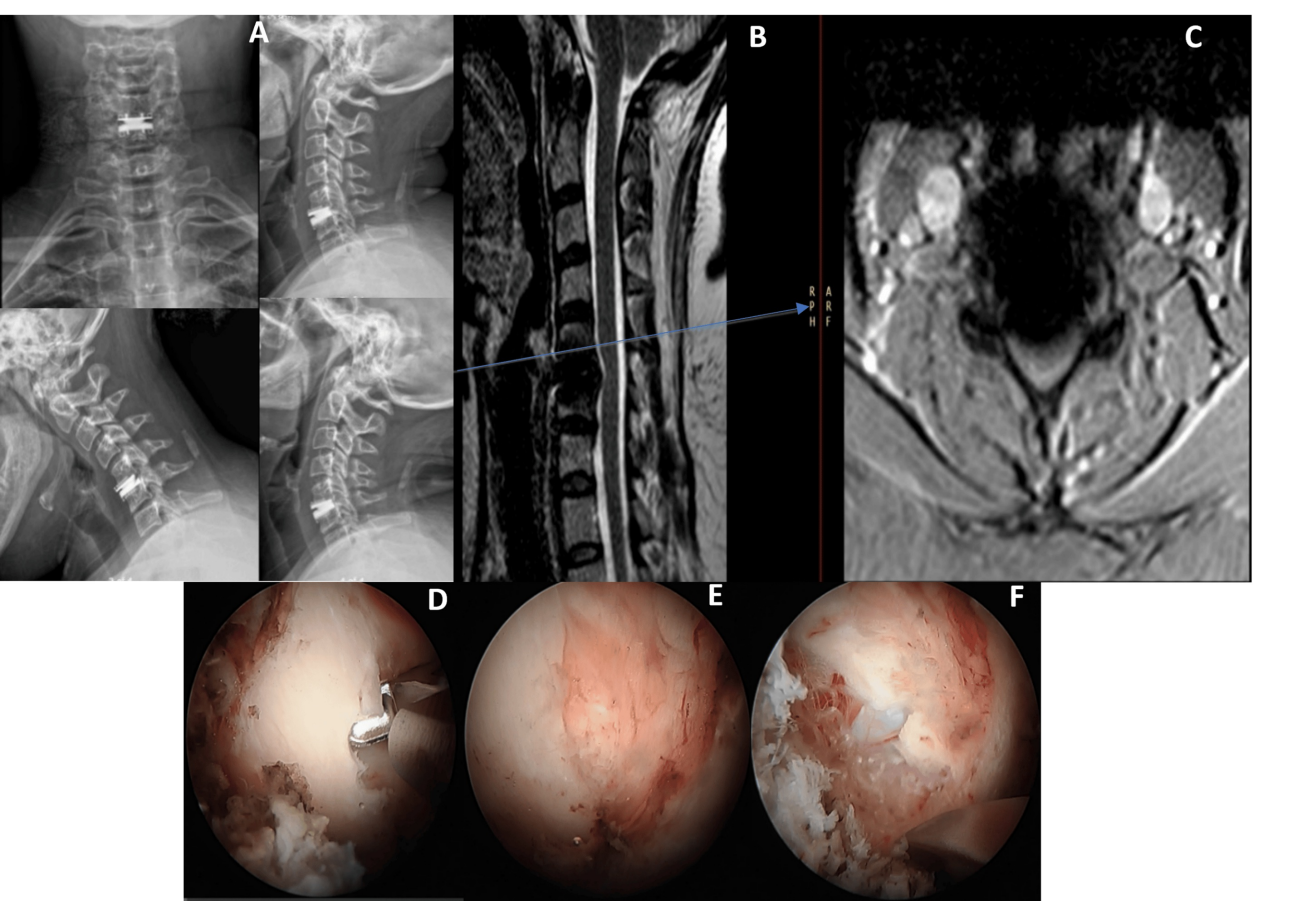
Cervical spine revision surgery in Case 1. (A) Preoperative anteroposterior, lateral, and dynamic cervical X-rays, status post C5/C6 TDR, showing TDR displacement with no postoperative improvement and persistent brachalgia. (B) Axial and sagittal T2-weighted cervical MRI demonstrating artifacts from the TDR, correlating with her persistent symptoms. (C) Intraoperative image following right posterior cervical C5/C6 decompression and discectomy, showing a laminotomy performed after initial TDR failed to alleviate symptoms. (D) Intraoperative image depicting the V point between the C5 and C6 lamina after decompression surgery. (E) Intraoperative image showing the decompressed nerve root with bleeding controlled using Surgicel (Ethicon, Inc., Raritan, New Jersey, United States) following posterior cervical decompression and discectomy. TDR: total disc replacement

Under general anesthesia, a targeted posterior decompression was performed endoscopically, minimizing disruption to surrounding tissues. The procedure did not involve disc-space distraction or manipulation of the prosthesis, thereby minimizing the theoretical risk of further anterior migration. Postoperatively, the patient experienced immediate and significant clinical improvement, with her VAS score decreasing from 8/10 to 0/10 and complete resolution of radicular symptoms. She reported no new anterior neck symptoms such as dysphagia or dysphonia, and her neurological status remained stable. Figure 1 shows the preoperative imaging limitations, intraoperative decompression views, and the postoperative anatomy following posterior UBE decompression.

The patient continued follow-up care locally abroad. The sustained clinical improvement and functional recovery documented at the two-year follow-up support the effectiveness of posterior UBE decompression in carefully selected revision cases, even in the absence of radiological follow-up. 

Case 2: Lumbar Spine Revision Surgery

A 49-year-old male patient presented with severe lower back pain and left leg pain (VAS 9/10) following an L5/S1 discectomy and TLIF performed one year prior. Despite the initial surgery, the patient experienced persistent symptoms that worsened over time, culminating in left foot drop (0/5), incontinence, and intractable pain, indicative of cauda equina syndrome. Diagnostic imaging revealed posterior displacement of the interbody cage at L5/S1, with MRI confirming compression of the thecal sac and nerve roots.

Due to the failure of the initial surgery and the risks associated with traditional open revision procedures, a minimally invasive approach using the UBE technique was recommended. The patient consented to undergo endoscopic UBE procedures, including removal of the displaced cage, removal of unilateral fixation, and endoscopic TLIF with a dual-direction expandable cage and percutaneous transpedicular screw fixation.

**Figure 2:**
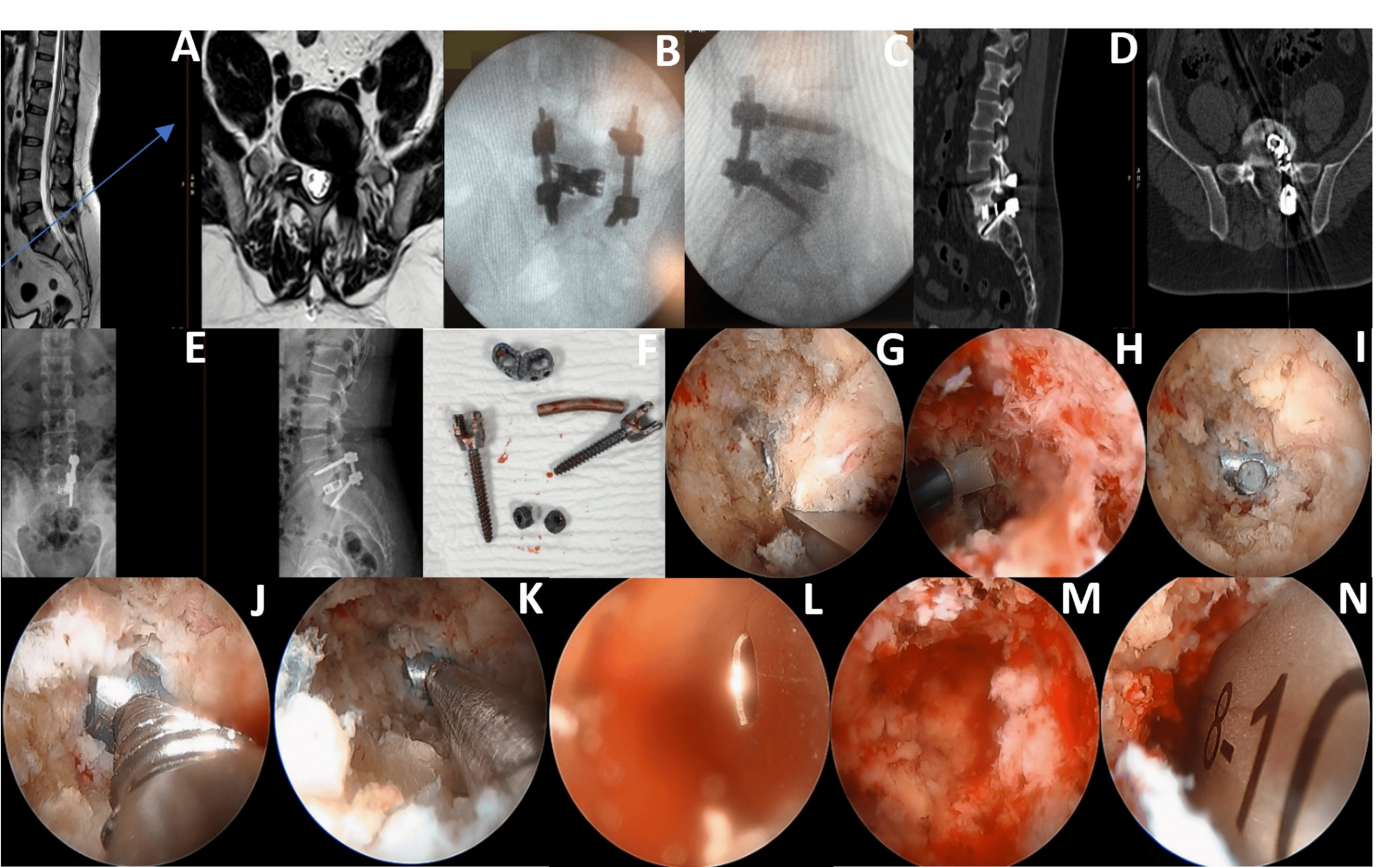
Lumbar spine revision with endoscopic TLIF in Case 2. (A) Axial and sagittal T2-weighted MRI images of the lumbar spine showing severe compression at the L5/S1 level caused by cage displacement from a previous TLIF surgery, compressing the thecal sac and nerve root. (B) AP intraoperative X-ray showing correct positioning of the cage and screws after left endoscopic removal of the malpositioned cage and screws, followed by left endoscopic TLIF using a dual-direction expandable cage. (C) Lateral intraoperative X-ray confirming the appropriate positioning of the cage and screws post-surgery. (D) Preoperative axial and sagittal CT scans displaying the displaced cage compressing the thecal sac and nerve root at L5/S1. (E) Preoperative AP and lateral X-rays illustrate unilateral screw fixation on the left side at L5/S1, with the displaced cage causing compression of the thecal sac and nerve root. (F) The removed cage and screws post surgery. (G) Intraoperative image showing the endoscopic removal of an osteophyte during the procedure. (H) Intraoperative image capturing the endoscopic insertion of the new expandable cage. (I) Intraoperative image showing the exposure of the previous TLIF cage. (J) Intraoperative image demonstrating an endoscopic trial connection of the cage holder to the previous TLIF cage. (K) Intraoperative image showing the successful connection of the TLIF cage holder to the previous TLIF cage. (L) Intraoperative image illustrating the endoscopic view. insertion of the new cage using a cage guide. (M) Intraoperative image depicting the endoscopic view after removing the previous TLIF cage. (N) Intraoperative image showing the endoscopic insertion of a cage trial during the procedure. TLIF: transforaminal lumbar interbody fusion; AP: anteroposterior

During the procedure, the UBE technique enabled effective visualization and removal of the posteriorly displaced cage, followed by the removal of unilateral screw fixation. The endoscopic TLIF approach facilitated the insertion of a dual-direction expandable cage and percutaneous screw fixation at L5/S1 with minimal disruption to the surrounding tissues. Figure 2 illustrates the severe cage displacement at L5/S1, intraoperative steps of cage removal and endoscopic TLIF, and the final implant positioning.

Postoperatively, the patient exhibited marked improvement, with muscle strength in the left foot increasing from 0/5 to 4/5. The VAS score for back and leg pain decreased from 9/10 to 1/10, reflecting substantial pain relief. Six months post surgery, the patient reported sustained improvements in both neurological function and pain levels, significantly enhancing his overall quality of life. 

Case 3: Complex Thoracolumbar Pathology in an Elderly Patient

A 71-year-old female patient with multiple significant comorbidities, including congestive heart failure (ejection fraction 30%), osteoporosis, diabetes, and hypertension, presented with severe mid and lower back pain, profound motor weakness (0-1/5), and sensory loss. She was unable to walk, and her pain severely limited her activities of daily living. Preoperatively, her VAS score was 7/10. MRI revealed pathological fractures at T10 with associated spinal cord compression and additional involvement at L3, findings consistent with her neurological presentation and indicating the need for urgent surgical intervention. Although T2 short tau inversion recovery (STIR) was not performed, the clinical and radiological features were strongly suggestive of an acute fracture at this level, guiding the decision for stabilization.

**Figure 3:**
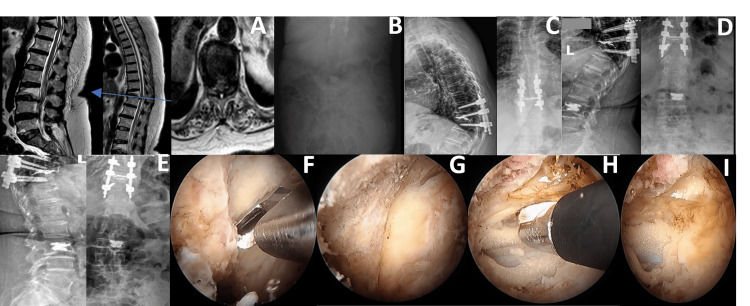
Thoracolumbar decompression and stabilization in Case 3. (A) Axial and sagittal T2-weighted MRI showing a pathological fracture at T10 with cord compression and another pathological fracture at L3. (B) AP abdominal X-ray taken on postoperative day 3, showing signs of ileus. (C) AP and lateral spine X-rays taken nine months postoperatively showed screws in good position and successful kyphoplasty after the patient underwent endoscopic decompression of D10 using a biportal endoscopic technique, ligamentotaxis, D10 kyphoplasty, and transpedicular screw fixation at D9, D10, and D11 bilaterally, and L3 kyphoplasty. (D) AP and lateral spine X-rays taken five months postoperatively showed screws in good position and successful kyphoplasty following the same procedure. (E) AP and lateral spine X-rays were taken on postoperative day 2, showing screws in good position and successful kyphoplasty after the same surgical intervention. (F) Intraoperative image showing a unilateral D10 laminectomy. (G) Intraoperative image showing the D10-D11 interlaminar space during the same procedure. (H) Intraoperative image showing contralateral decompression during the same surgical intervention. (I) An intraoperative image shows excellent cord decompression following the procedure AP: anteroposterior

Given her high anesthetic risk and complex medical background, a multidisciplinary team elected to pursue a minimally invasive approach using UBE under local anesthesia with sedation. This approach aimed to minimize physiological stress while still achieving adequate decompression and stabilization. Endoscopic decompression of the spinal cord at T10 was performed with minimal disruption to surrounding tissues, followed by balloon kyphoplasty at T10 and L3 to restore vertebral height and address instability. Bilateral transpedicular screw fixation at D9, D10, and D11 provided additional structural support. Figure 3 presents key MRI findings, postoperative radiographs, and intraoperative endoscopic decompression views from the thoracolumbar reconstruction.

The procedure lasted 155 minutes. While this duration is relatively long for a patient with significant comorbidities, it was tolerated without major intraoperative complications. We acknowledge that prolonged surgical time in the prone position under sedation carries inherent risks, including potential airway compromise and increased anesthetic requirements, which may have contributed to the patient’s postoperative ileus. It is also possible that similar surgical durations under general anesthesia could mitigate some of these risks, and that more extensive correction of kyphotic angulation might have been achievable with an open procedure. 

Despite these limitations, the patient experienced significant postoperative improvement. She began ambulating with a walker on the first postoperative day and progressed to independent walking with minimal pain (VAS 0-1/10) within one month. At the two-year follow-up, she maintained excellent functional outcomes and independence in daily activities.

## Discussion

Revision spine surgery remains one of the most technically demanding areas of spinal practice due to the presence of altered anatomy, dense scar tissue, and potential complications such as dural tears, infection, previous instrumentation, and neurovascular injury. Careful evaluation of the patient’s clinical presentation, imaging findings, and the relative risks and benefits of different surgical approaches is essential in guiding decision-making. Complication rates in revision spine surgery can reach 25-30%, underscoring the need for approaches that minimize surgical morbidity [22].

In this context, minimally invasive techniques such as UBE spine surgery have emerged as promising alternatives, particularly in complex and revision cases. UBE enables targeted decompression with reduced tissue disruption, potentially lowering perioperative morbidity and shortening recovery time. These advantages become especially relevant in revision surgeries where scar tissue and distorted anatomy increase the difficulty and risks of traditional open approaches.

Several studies have highlighted the utility of UBE in challenging revision scenarios. Choi et al. described its application in recurrent lumbar disc herniation (RLDH), demonstrating faster postoperative pain relief and earlier functional recovery compared with microscopic techniques [23]. Kim reported the effectiveness of UBE-assisted removal of bony fragments in revision lumbar surgery, contributing to improved neurological recovery and pain reduction [24]. Wang et al. showed that the use of a dual-direction expandable cage facilitated stabilization in revision arthrodesis for unstable adjacent segment disease, further supporting the versatility of UBE in complex lumbar cases [25].

Our first case illustrates the feasibility of UBE in cervical revision surgery, achieving complete resolution of radicular pain (VAS 8/10 → 0/10) and sustained clinical improvement at two-year follow-up. While most literature on UBE focuses on lumbar pathology, our findings suggest that this approach can also be successfully adapted for complex cervical revisions.

Case 2 demonstrated outcomes consistent with those reported by Kim, including muscle strength recovery and pain reduction following UBE-assisted revision [24]. These findings emphasize the potential role of endoscopic techniques in managing revision cases where altered anatomy and prior instrumentation would otherwise complicate standard surgical approaches.

Case 3 further highlights the applicability of UBE in patients with significant comorbidities, where the reduced invasiveness of the approach may offer important advantages. A 71-year-old patient with severe cardiac dysfunction and osteoporosis, who was bedridden preoperatively, regained independent ambulation following UBE decompression combined with balloon kyphoplasty and limited fixation. Hoe et al. have similarly reported that UBE can minimize postoperative facet joint damage and reduce pain [26].

This case illustrates that UBE can offer a viable alternative for high-risk patients, enabling decompression and stabilization with minimal soft tissue disruption. However, it also highlights important limitations, including longer operative time, anesthetic considerations, and potential trade-offs in deformity correction compared to open surgery. Recognizing these limitations is essential in understanding that endoscopic approaches complement rather than replace traditional open techniques, and careful patient selection remains critical. Although kyphoplasty restored vertebral height and alleviated pain, its capacity to prevent fracture-related instability was limited, and additional pedicle screw fixation was required. Postoperative imaging revealed a mild increase in kyphotic angulation at T10 and L3, raising questions about the adequacy of stabilization in multi-level fractures. These findings emphasize the need for further biomechanical research and comparative studies to evaluate whether extended fixation or alternative stabilization strategies yield improved long-term outcomes.

It is important to acknowledge that traditional open surgery continues to offer distinct advantages, particularly when extensive decompression or robust stabilization is required. Open approaches provide direct visualization and access to a wide surgical field, which may be necessary in certain complex pathologies. UBE should therefore be viewed not as a replacement for open techniques but as a complementary option within the surgical armamentarium, one that can offer significant benefits in selected patients by reducing soft tissue disruption, blood loss, and recovery time.

Overall, our experience demonstrates that UBE can be an effective tool in the management of challenging and revision spinal pathologies across cervical, thoracic, and lumbar regions. Yet, its limitations, including a steep learning curve, potentially prolonged operative times, and constraints in achieving deformity correction, must be recognized. Future prospective, controlled studies with larger cohorts are needed to validate these findings, clarify patient selection criteria, and define the optimal role of UBE relative to conventional open approaches in complex spinal surgery.

The UBE technique shows considerable potential in managing spinal disorders across cervical, thoracic, and lumbar levels while maintaining minimal invasiveness and encouraging clinical outcomes. Our experience supports its feasibility even in complex and revision cases; however, this case series has several limitations. Neurological function was assessed clinically, but detailed sensory evaluations were not systematically documented in all patients. The absence of a control group limits direct comparison with conventional open surgery, and the small sample size reduces the generalizability of our observations. As a retrospective case series, the possibility of selection bias also exists. Additionally, the mild increase in kyphotic angulation observed in Case 3 underscores the need for further biomechanical investigation. Larger, prospective, controlled studies are required to validate these findings and help establish standardized protocols for UBE application.

## Conclusions

UBE spine surgery is a highly effective and minimally invasive technique for managing complex and revision spinal cases. The versatility of UBE in addressing a wide range of spinal pathologies, combined with its ability to be performed under local anesthesia, offers a promising alternative to traditional open surgeries. The cases presented in this series highlight the potential of UBE to provide excellent clinical outcomes with minimal invasiveness, making it a game-changing approach in the management of challenging spinal conditions. Future studies and long-term follow-ups are warranted to further validate these findings and explore the broader applications of UBE in spine surgery.
